# Iron-based microbial interactions: the role of iron metabolism in the cheese ecosystem

**DOI:** 10.1128/jb.00539-24

**Published:** 2025-04-16

**Authors:** Rina Mekuli, Mahtab Shoukat, Eric Dugat-Bony, Pascal Bonnarme, Sophie Landaud, Dominique Swennen, Vincent Hervé

**Affiliations:** 1 Université Paris-Saclay, INRAE, AgroParisTech, UMR SayFood 84338 , Palaiseau, France; Geisel School of Medicine at Dartmouth, Hanover, New Hampshire, USA

**Keywords:** nutrient, metal, fermented food, mineral, dairy product, siderophores, ferrous iron, ferric iron

## Abstract

Iron is involved in various microbial metabolisms and interactions and is an essential micronutrient for most microorganisms. This review focuses on the cheese ecosystem, in which iron is sparse (median concentration of 2.9 mg/kg based on a literature survey) and of limited bioavailability due to the presence of various metal-binding agents in the cheese matrix. Cheese microorganisms overcome this low bioavailability of iron by producing and/or importing ferric iron-specific chelators called siderophores. We introduce these siderophores and their specific transporters, which play a key role in ecological interactions and microbial metabolism. We discuss the impact of iron on all the major taxa (fungi, bacteria, and viruses) and functional groups (starters, ripening microorganisms, and pathogens) present and interacting in cheese, from the community to individual levels. We describe the ways in which cheese-ripening microorganisms use iron and the effects of iron limitation on major metabolic pathways, including the tricarboxylic acid (TCA) cycle and amino-acid biosynthesis. The cheese ecosystem is a relevant *in situ* model for improving our understanding of iron biochemistry and its putative role in microbe–microbe interactions. Yet, this review highlights critical gaps in our understanding of iron’s role in cheese from fundamental ecological and biochemical perspectives to applied microbiology, with broader implications for the quality, safety, and organoleptic properties of cheese.

## CHEESE AND ITS MICROBIOME

Cheese is one of the oldest and most popular fermented dairy products worldwide (1–3). Cheeses form a group of fermented milk-based food products with diverse sensory features (4, 5). They consist principally of water, lipids, proteins, carbohydrates, and trace minerals, the final composition depending on the type of cheese and the process used to produce it (6). Cheese constitutes a complex and dynamic ecosystem resulting from a series of biological and chemical processes occurring between fermentation and ripening and driven by a succession of diverse microorganisms (7–9). There are five successive steps in the cheese-making process: (i) milk acidification; (ii) coagulation; (iii) cutting, cooking, stirring, pressing, and other processes that promote syneresis (i.e., the release of the whey); (iv) molding; and (v) salting (10). Once these main steps have been completed (i.e., milk acidification and coagulation), the cheese curd can be ripened to further enhance and modify the flavor and texture of the cheese (11).

The cheese microbiota plays a key role in cheese-making, and the component microorganisms are of two main types: starters, the microorganisms added by the cheesemaker, and the endogenous microorganisms naturally present in the milk and the cheese environment (12). In addition, adventitious, spoilage, and pathogenic microorganisms may also be present (13, 14).

Taxonomically, the cheese microbiota consists mostly of bacteria, including lactic acid bacteria (LAB) (15, 16) and ripening bacteria (17, 18), together with fungi, including yeasts (19) and filamentous fungi (20). To date, no archaea have ever been identified in cheese. A decade ago, a review identified 104 bacterial and 39 fungal genera present in cheese rind: 46 *Pseudomonadota*, 28 *Actinomycetota*, 24 *Bacillota*, 5 *Bacteroidota*, 1 *Acidobacteriota*, 21 molds, and 18 yeasts (12). More recently, Irlinger *et al.* identified a total of 273 fungal species from 136 genera and 810 bacterial species from 285 genera in a large-scale study of French cheeses with a protected designation of origin (18). Traditional smear-ripened cheeses host diverse bacteria, including species belonging to the genera *Glutamicibacter*, *Brevibacterium*, *Corynebacterium*, *Microbacterium*, *Staphylococcus*, *Halomonas*, *Pseudoalteromonas*, *Psychrobacter*, *Pseudomonas*, and *Psychroflexus*. They also contain *Geotrichum candidum*, *Debaromyces hansenii*, *Saccharomyces cerevisiae*, *Yarrowia lipolytica*, *Kluyveromyces* spp., *Torulaspora delbrueckii*, and *Candida* spp. among the most dominant fungal taxa (21, 22). The cheese ecosystem also contains viruses. Bacteriophages are increasingly being studied in this ecosystem (23), but little is known about the mycoviruses potentially present (24).

**TABLE 1:** *In silico* prediction of BGCs encoding siderophores in the genomes of bacteria with different functions in cheese^*a*^*^,^*^*b*^

Taxon	Ecology	Genome sequence	Siderophore	Similarity (%)
*Arthrobacter bussei* KR32	Ripening	GCF_009377195.2	Desferrioxamine	66
*Bacillus smithii* DSM 4216		GCF_001050115.1	Schizokinen	70
*Brachybacterium alimentarium* 908_11	Ripening	GCF_002332425.1	FW0622	37
*Brachybacterium tyrofermentans* FME25	Ripening	GCF_014897585.1	FW0622	37
*Brevibacterium aurantiacum* ATCC 9175	Ripening	GCF_900169065.1	Legonoxamine	33
*Brevibacterium casei* CIP 102111	Ripening	GCF_900169275.1	FW0622	25
*Corynebacterium variabile* DSM 44702	Ripening	GCF_000179395.2	Madurastatin	23
*Glutamicibacter arilaitensis* Re117	Ripening	GCF_000197735.1	Desferrioxamine	50
*Hafnia alvei* GB001	Ripening	GCF_900095695.1	Desferrioxamine E	75
*Halomonas nigrificans* DSM 105749	Ripening	GCF_002374315.1	Potashchelin	36
			Potashchelin	48
			Potashchelin	9
*Luteococcus japonicus* LSP Lj1	Ripening	GCF_900163845.1	Benarthin/dibenarthin	38
*Mammaliicoccus fleurettii* DSM 13212	Spoilage/pathogenic	GCF_003970575.1	NI siderophore	NA
*Microbacterium gubbeenense* DSM 15944	Ripening	GCF_000422745.1	Fuscachelin	66
*Micrococcus luteus* Mu201	Ripening	GCF_900163885.1	NI siderophore	NA
*Pseudoalteromonas nigrifaciens* FME68	Ripening	GCF_014897925.1	Desferrioxamine E	75
*Pseudoalteromonas prydzensis* FME14	Ripening	GCF_014861445.1	Variochelin	20
*Pseudomonas carnis* B157	Spoilage/pathogenic	GCF_019570475.1	Azotobactin D	50
			Pyoverdine SMX-1	12
			Mevalagmapeptide	4
			Pf-5 pyoverdine	11
*Pseudomonas fluorescens* ITEM 17298	Spoilage/pathogenic	GCF_002319065.1	Pyoverdine SMX-1	12
			Pyoverdine SMX-1	19
			Pf-5 pyoverdine	19
			Azotobactin	50
			Mevalagmapeptide	4
*Salmonella enterica* subsp. *enterica* serovar Dublin	Spoilage/pathogenic	GCF_900608935.1	Enterobactin	100
*Staphylococcus aureus* ProNaCC-02	Spoilage/pathogenic	GCA_033753855.1	Staphyloferrin A	100
			Staphyloferrin B	100
			Staphylopine	100
			Staphyloferrin B	100
*Staphylococcus casei* DSM 15096	Ripening	GCF_030294405.1	Staphyloferrin A	100
*Staphylococcus equorum* DSM 15097	Ripening	GCF_002901955.1	Staphyloferrin B	100
			Staphyloferrin A	75
*Staphylococcus saprophyticus* 429A	Ripening	GCF_001747585.1	Staphyloferrin A	100
*Vibrio casei* DSM 22364	Ripening	GCF_002218025.2	Aerobactin	62

^*a*^ The selected genomes are derived from bacteria isolated from cheese, based on a comprehensive literature review. Siderophore BGCs were found in 24 of the 63 genomes analyzed. BGCs were detected and identified with antiSMASH v.7.1.0 (25) (see supplemental material for details and data).

^*b*^ BGC, biosynthetic gene cluster; NA, not available.

The interactions between these diverse microbial communities play a key role in shaping the composition and dynamics of the entire cheese microbiome, ultimately affecting cheese safety and quality (26–28). Strain-level variations across the various cheese communities may contribute to variations in the quality of surface-ripened cheeses (29), and abiotic factors also modify community composition and ecosystem functioning. Indeed, a recent synthesis identified the origin of the milk, the pasteurization process, geographic location, and type of cheese as the main drivers of cheese microbial community composition and diversity, contributing to about 65% of the variation of microbial community composition worldwide (30). Another national study identified human drivers, dairy species, and geographic distance as the main factors shaping the cheese microbiota (18).

There are two main types of cheese-ripening process, conferring different features on the final product: i.e., surface-ripened cheeses and internally ripened cheeses. The surface-ripened cheese group includes mold-ripened and smear-ripened cheeses (31). Smear-ripened cheeses have a distinct viscous layer with a red-orange smear covering the surface, mainly due to bacterial activity. Some yeasts can also modulate the color development of ripening bacteria through their deacidification activity (32). By contrast, mold-ripened cheeses have thick layers of fungi, such as *Geotrichum candidum* or *Penicillium camemberti* (33) on their surfaces.

The development of the smear on surface-ripened cheese depends primarily on ripening conditions, i.e., cheese surface pH, relative humidity (RH), ripening temperature, salt concentration, frequency of rind washing, and ripening period (34). The contribution of these abiotic factors to the development of microbial communities is well documented (35). Surface moisture and pH are critical factors shaping the composition of the rind of bacterial-ripened, mold-ripened, and natural-rind cheeses. Ripening temperature is another important abiotic factor controlling the colonization of the cheese by cheese-ripening bacteria and yeasts (e.g., optimal range of 10°C–15°C for smear-ripened cheeses) (36). Cheese ripening is associated with changes in pH. At the start of the ripening stage, the pH of the cheese lies between 4 and 5 due to the activity of LAB. This low pH favors the growth of aerobic molds and yeasts. These fungi increase the pH at the surface of the cheese by transforming lactate into CO_2_ and amino acids into ammonia (37). When the pH is greater than 5.5, surface-ripening bacteria begin to colonize the cheese. At the end of the ripening process, the pH is about 8 (38). RH is also crucial as there must be sufficient moisture to ensure that the surface does not dry out completely, enabling the ripening microbiota to grow. The microbes at the surface of the cheese require a mean RH of *>*90% (33). Salt level is another crucial technological factor with an impact on the growth of various bacterial strains from surface-ripened cheeses. Generally, surface-ripened cheeses contain 1.5%–2.3% NaCl (12). Indeed, lower salt levels may allow spoilage microorganisms to proliferate, as demonstrated in a previous study (39) showing significant growth of the food spoilage bacterium *Pseudomonas fragi* at an NaCl concentration of 1.3%. However, while the effects and importance of these abiotic factors have been documented for years, less is known about the effect of nutrients, including metals, on the composition and metabolism of the cheese microbial community.

## SCARCITY OF IRON IN CHEESE

**Fig 1:**
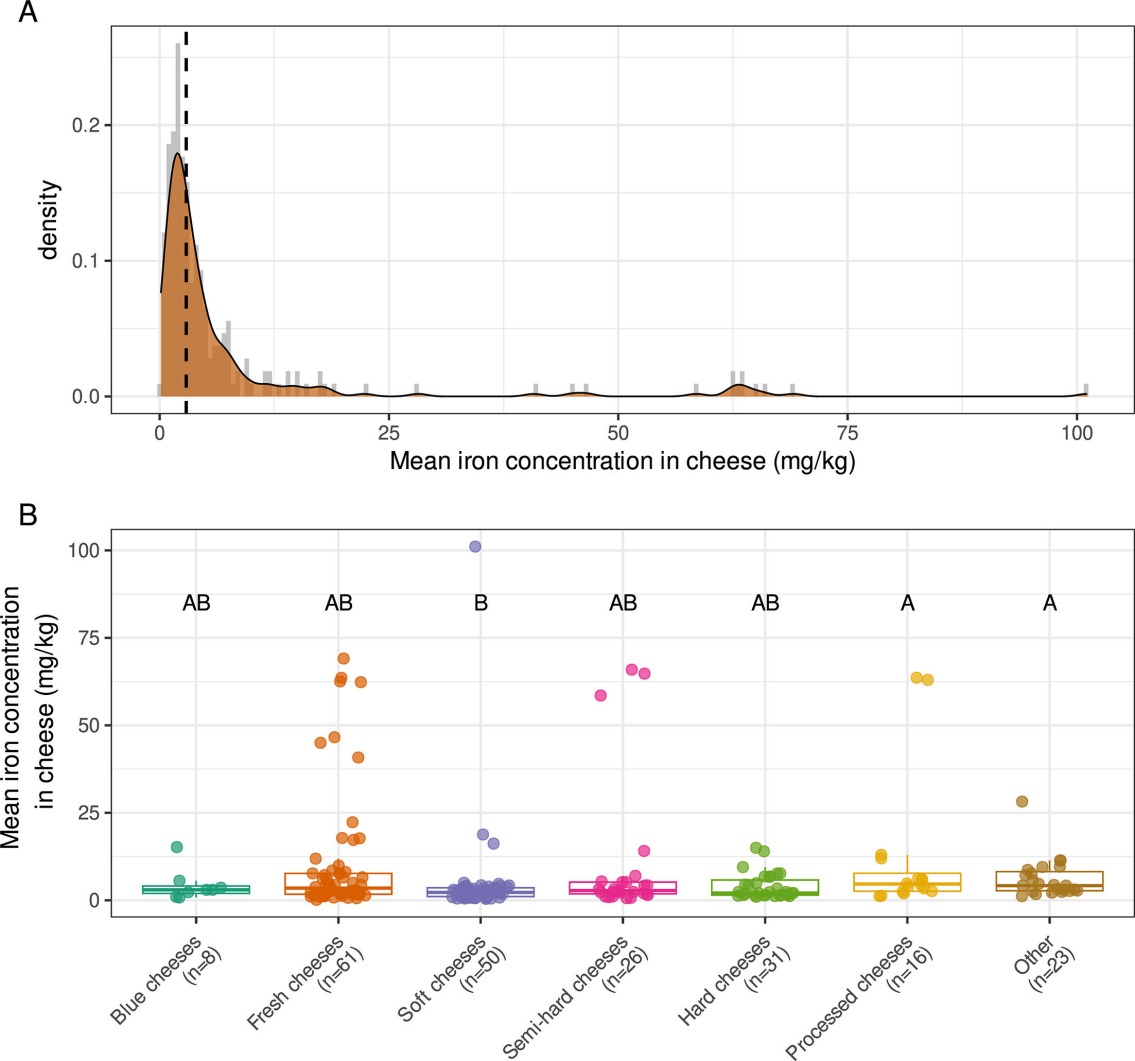
Iron concentrations (mg/kg) in cheese according to a literature survey of 21 research articles and 2 food databases. (**A**) Density plot of the 215 mean iron concentrations extracted from the literature survey. The vertical dashed line indicates the overall median concentration (2.9 mg/kg). (**B**) Mean iron concentration in the different technological families of cheese. Statistical comparisons were performed with the Kruskal–Wallis test followed by Dunn’s test with Benjamini–Hochberg correction.

Iron is scarce in cheeses for two main reasons: the concentration of iron is naturally low in milk, and iron bioavailability is low due to the presence of proteins and other macromolecules that sequester iron. Indeed, cheese is a medium in which iron levels are exceptionally limiting (Fig. 1) due to the very low iron content of milk, e.g*.,* 0.09–0.90 mg/kg for cow’s milk (40). By comparison, total iron content is much higher at about 20 mg/kg in beef meat (41) and about 180 mg/kg in human feces (42), and the median concentration of iron in soil is about 25,000 mg/kg (43, 44). Iron is evenly distributed throughout the milk in whey protein [consisting principally of beta-lactoglobulin, alpha-lactalbumin, bovine serum albumin, and immunoglobulins (45)], casein, and fat globules, each of which binds large proportions of iron—i.e., 29%, 24%, and 14%, respectively—whereas 32% of iron is bound to a low-molecular-weight fraction that is probably more bioavailable and includes small organic acids, lactose, and nutrients (40). The globular glycoproteins lactoferrin and transferrin are the two main proteins chelating iron in milk and cheese. Lactoferrin, which has two iron-binding sites for Fe^3+^, is present in large amounts (20–200 mg/mL). By contrast, transferrin is present in only trace amounts in cow’s milk (0.01–0.1 mg/mL) (46). The lactoferrin concentration in cheese depends principally on the cheese-making process. For instance, thermal treatments, such as milk pasteurization, lead to a partial hydrolysis of lactoferrin, and ultra-high temperature treatment may result in the complete denaturation of this protein (46). Cow’s milk caseins bind iron with high affinity via clusters of phosphoserine residues. One study performed with sodium caseinate identified 14 ferrous iron-binding sites on caseins. Moreover, the binding of iron to caseins is relatively robust to changes in pH and ionic strength (47).

We performed a quantitative assessment of iron concentrations in cheese based on a literature survey. We retained 21 research articles and two food databases, from which we extracted 215 mean concentrations. The overall median concentration of iron was 2.9 mg/kg (Fig. 1), confirming that cheese is a food with a low iron content (48). Surprisingly, cheese technology does not seem to have a strong impact on iron concentration (Kruskal–Wallis rank-sum test, *P* = 0.0112). Indeed, for the six technological families considered, we found only that processed cheeses had significantly higher iron concentrations than soft cheeses (corrected Dunn’s test, *P* = 0.021).

Iron fortification, i.e., a process involving the addition of iron to a food matrix to increase nutritive value (49), is difficult to achieve in cheese due to the ability of iron to replace other divalent cations in the milk system (50). The most common added sources of iron are iron salts, such as chloride and sulfate. These iron salts have a high solubility in both water and milk but may interact freely with the components of milk, potentially changing its sensory characteristics (51). The chemical form of iron determines the distribution of the iron in milk. Iron salts, such as sulfate, chloride, and nitriloacetate readily donate iron, modifying the structure of casein. Conversely, iron chelators and iron-binding proteins, such as lactoferrin, ferritin, and ethylenediaminetetraacetic acid, bind iron strongly and do not, therefore, alter the colloidal phase (51).

## PHYSICOCHEMICAL PROPERTIES OF IRON

Iron is an essential micronutrient for almost all microorganisms as it is required for their metabolic and biological functions (see below). However, it is frequently limiting for microbial growth under aerobic conditions due to its poor solubility and stability. It can also be toxic for living cells in aerobic conditions because of its ability to catalyze the production of reactive oxygen species (ROS) via the Haber–Weiss/Fenton reaction. These ROS can damage biological molecules (e.g., carbohydrates, nucleic acid, lipids, and amino acids) (52). The oxidation state and concentration of iron must therefore be carefully controlled in biological systems.

In nature, iron is commonly found in two oxidation states: the ferrous Fe^2+^ and ferric Fe^3+^ forms. Fe^2+^ is water soluble, whereas Fe^3+^ is relatively insoluble at physiological pH. In aqueous solution and at pH 7, the concentration of free Fe^3+^ has been estimated at 1.4 × 10^−9^ mol/L (53), below the level required for the growth of aerobic microorganisms. The pH is the principal factor controlling the solubility of iron ions in aqueous solution. The main reason for the low bioavailability of iron is its poor solubility at neutral pH. The bioavailability of iron is higher at low pH due to the greater stability and solubility of iron in acidic conditions. One study reported that a pH of <3 greatly stabilizes ferrous iron and significantly increases the solubility of ferric iron relative to neutral pH (54). Furthermore, the oxidation of ferrous to ferric iron occurs much more slowly in acidic conditions because low pH confers a more reducing environment favoring Fe^2+^.

## IRON ACQUISITION

Aerobic bacteria are highly dependent on iron. They have therefore developed several iron uptake mechanisms to ensure that they obtain sufficient amounts of this nutrient for their survival. This review focuses on siderophore-mediated iron acquisition due to the physicochemical properties of the cheese surface (55, 56). Siderophores are a group of structurally diverse, low-molecular-weight compounds synthesized by microbes to sequester iron from the environment. They have a high affinity for Fe^3+^, allowing them to capture these ions from lactoferrin (57).

**Fig 2:**
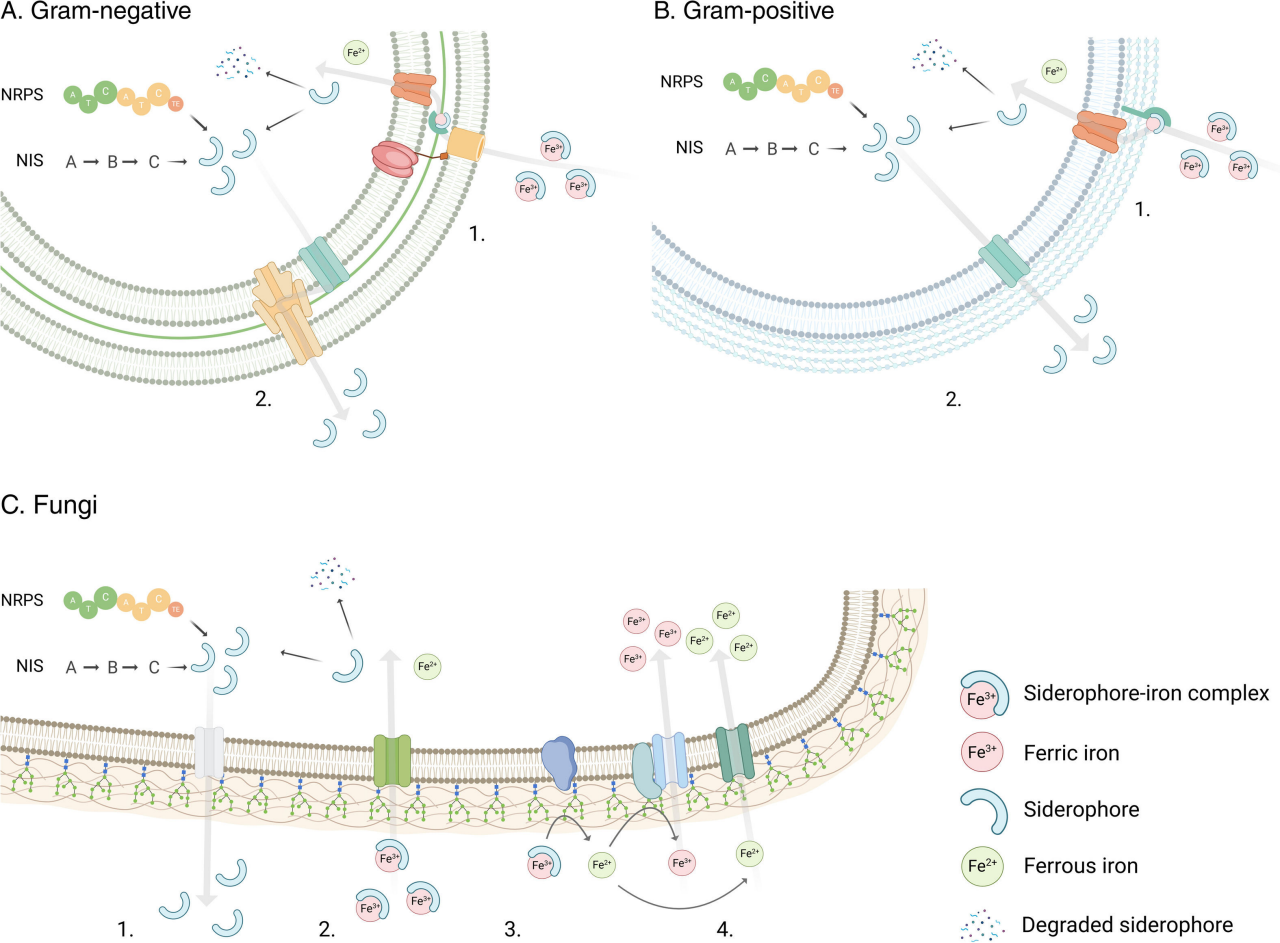
Siderophore-based iron transport systems in bacteria and fungi. (A1) In Gram-negative bacteria, the siderophore-iron complex is transported across the outer membrane via a TonB-dependent mechanism, with the energy for transport acquired by the TonB-ExbB-ExbD complex. A periplasmic binding protein binds to the siderophore-iron complex and is transported into the cytoplasm by a specific ABC transporter (56). Alternatively, some bacteria release iron from the siderophore in the periplasm, as is the case for pyoverdines (63). (A2) Mechanisms for exporting unbound siderophores are less well understood. In *Escherichia coli*, exporters from the major facilitator superfamily (MFS) transport the siderophore to the periplasm; it is then secreted by the resistance nodulation cell division and TolC efflux system (64). (B1) Gram-positive bacteria, which lack the outer membrane, use a substrate-binding protein anchored to the membrane for siderophore binding, and the siderophore is then taken up by an ABC transporter (56). (B2) In Gram-positive bacteria, siderophores are probably exported by MFS-type efflux pumps (65). (C1) Little is known about siderophore export in fungi. (C2) Siderophore transport in fungi is performed by SIT/ARN subfamily symporters from the MFS superfamily (66). (C3) Fungi use a reductive iron transport system in which ferric iron is reduced by FRE enzymes. Ferrous iron is then re-oxidized and transported via a multicopper ferroxidase (Fet3p) and a permease (Ftr1p) (67). (C4) Possible low-affinity ferrous iron transport following the reduction of iron by FRE (66). In both bacteria and fungi, the siderophores are subsequently either degraded or recycled and released from the cell. Created in https://BioRender.com.

There are two main pathways of siderophore synthesis: the non-ribosomal peptide synthetase (NRPS)-dependent and NRPS-independent siderophore (NIS) synthesis pathways (Fig. 2). The NRPS enzymes are modular proteins with catalytic domains that incorporate one amino acid at a time into peptides via several reactions. Siderophores produced through this pathway include enterobactin, yersiniabactin, vibriobactin, acinetobactin, pyoverdine, pyochelin, and mycobactin (58–60). By contrast, a single reaction is required for the production of NIS siderophores. All known NIS enzymes have a conserved IucA/IucC domain responsible for iron uptake. These enzymes are classified into three main groups: the A, B, and C types, specific for citric acid, α-ketoglutaric acid, and citric acid or succinic acid derivatives, respectively (61). A review considering hundreds of sequences with both the IucA and FhuF (ferric iron reductase) domains showed that most of the sequences were assigned to *Actinomycetota* (46.2%) and *Bacillota* (52.4%) (59). *Actinomycetota* encompass an abundant portion of the cheese rind population (28 genera). Depending on the moieties responsible for iron-binding, these siderophores can be classified as hydroxamate, catecholate, carboxylate, and mixed types (62). The diverse chemical structure of siderophores dictates their transport systems and substrate specificity.

The differences in cell wall composition between Gram-positive and Gram-negative bacteria result in their use of different iron transport mechanisms (Fig. 2). Gram-positive bacteria usually carry a tethered substrate-binding protein, which is transported to the cytosol via an inner membrane (IM) ABC transporter. Gram-negative bacteria have an additional outer membrane (OM) receptor and an energy-dependent TonB mechanism (56). The OM receptor generally has a higher specificity than the IM substrate-binding protein (68, 69). This level of knowledge about iron transport mechanisms is currently lacking for most ripening microorganisms. The model organism, *Escherichia coli*, has specific OM receptors for ferrichrome, aerobactin, rhododulurate, coprogen, and ferrioxamine (FhuA, IutA, FhuE, and FoxA, respectively), but all these substrates are transported into the cytosol by the binding protein FhuD (69, 70). Similarly, the FepB substrate-binding protein is responsible for transporting enterobactin, salmochelin, and monomeric catecholates (FepA, IroN, Fiu, and Cir) (71, 72).

Fungi can also synthesize NIS and non-ribosomal pep­tide synthetase (NPRS) siderophores, but their iron acquisition mechanisms differ from those of bacteria. A family of mannoproteins in the outer layer of the cell wall, the facilitator of iron transport proteins, retains siderophore-iron complexes in the cell wall (66). Fungi typically use two siderophore iron acquisition systems: a reductive system and a non-reductive system. These systems have been described in detail for *S. cerevisiae*. The reductive system involves ferric reductase enzymes (FREs), which reduce ferric iron, releasing the iron from the siderophore due to the low affinity of the siderophore for ferrous iron (73). The specificity of FREs is quite diverse: Fre1 and Fre2 can reduce both hydroxamates and catecholates; Fre3 reduces hydroxamates; and Fre4 is specific for rhodotorulic acid. Once the iron is reduced, it is re-oxidized and transported into the cell by a high-affinity complex consisting of a multicopper ferroxidase (Fet3p) and a permease (Ftr1p) (67). The reductive iron uptake mechanism is used if sufficient quantities of siderophores are available. If siderophores are scarce, non-reductive siderophore transport systems mediate iron uptake (66). The transporters involved are probably membrane potential-dependent proton symporters from the ARN/SIT subfamily displaying specificity for different groups of fungal and bacterial siderophores. In *S. cerevisiae*, Arn1p transports ferrichrome, ferrichrome-type siderophores, and coprogen. It also transports triacetylfusarinine C (TAFC) with lower efficiency. Arn2p/Taf1p transports only TAFC. Arn3p/Sit1p has a broader specificity, transporting ferrichromes, coprogen, and bacterial ferrioxamines. Arn4p/Enb1 transports the catcholate-type siderophore enterobactin (66, 74). The substrate specificities of these siderophores have also been experimentally demonstrated in *Candida albican*s, *Schizosaccharomyces pombe*, *Aspergillus nidulans*, and *Fusarium graminearum* (74).

The mechanisms of iron release from siderophores are less well understood. Siderophores have a high affinity for Fe^3+^. The reduction of the iron to Fe^2+^ therefore decreases the affinity of binding, leading to iron release and the secretion of the siderophore for re-use. Another strategy involves the hydrolysis of the siderophores to release the iron, but this mechanism has the disadvantage that the siderophores cannot be recycled directly (75).

**Fig 3:**
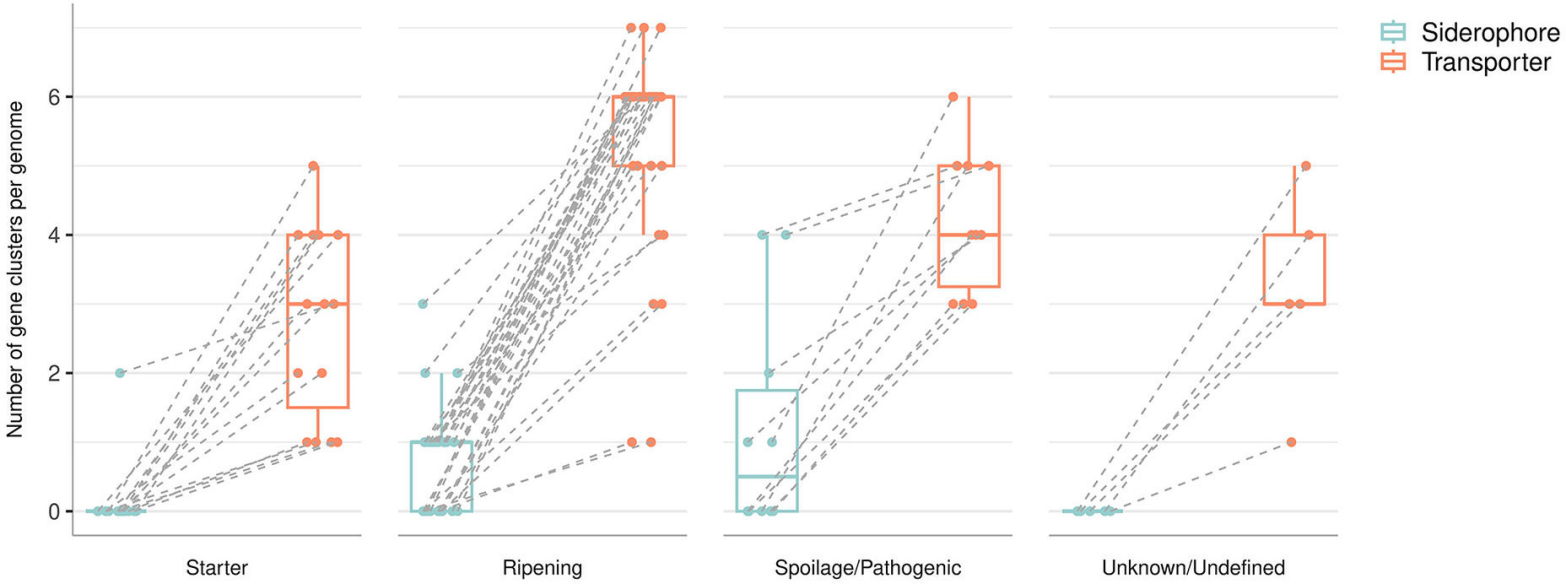
Number of gene clusters involved in siderophore synthesis and transport per genome in starter, surface-ripening, spoilage/pathogenic, and unknown/undefined bacteria. The selected genomes (*n* = 63) are derived from bacteria isolated from cheese, based on a comprehensive literature review. Genomic analyses were performed with antiSMASH v.7.1.0 (25) and the Transporter Automated Annotation Pipeline (80) (see supplemental material for details and data).

Siderophores from pathogenic microbes and from marine and soil environments have been extensively studied, but those produced by cheese-ripening microorganisms remain largely uncharacterized (76–78). We summarize what is currently known about cheese microorganisms and siderophore production in Table 1, based on an *in silico* analysis of 63 bacterial and 13 fungal genomes from cheese. Figure 3, based on data from Table 1 and the supplemental material, highlights the distinction between the bacterial biosynthetic gene clusters involved in siderophore biosynthesis and those involved in siderophore transport. Interestingly, all the bacterial genomes analyzed possess at least one siderophore transporter, whereas only 24 of the 63 genomes possess at least one biosynthetic gene cluster involved in siderophore biosynthesis. This suggests that a sizable proportion of the microbial community present in cheese can benefit from the presence of siderophores in the ecosystem without actually synthesizing them; they may therefore be considered “cheaters” (79). In particular, surface-ripening bacteria exhibit the largest numbers of siderophore transporters, highlighting their potential for involvement in iron-mediated interactions. An extensive table providing information about siderophores and their transporters is available in the supplemental material.

## ROLE OF IRON IN CHEESE MICROBIAL INTERACTIONS

**Fig 4:**
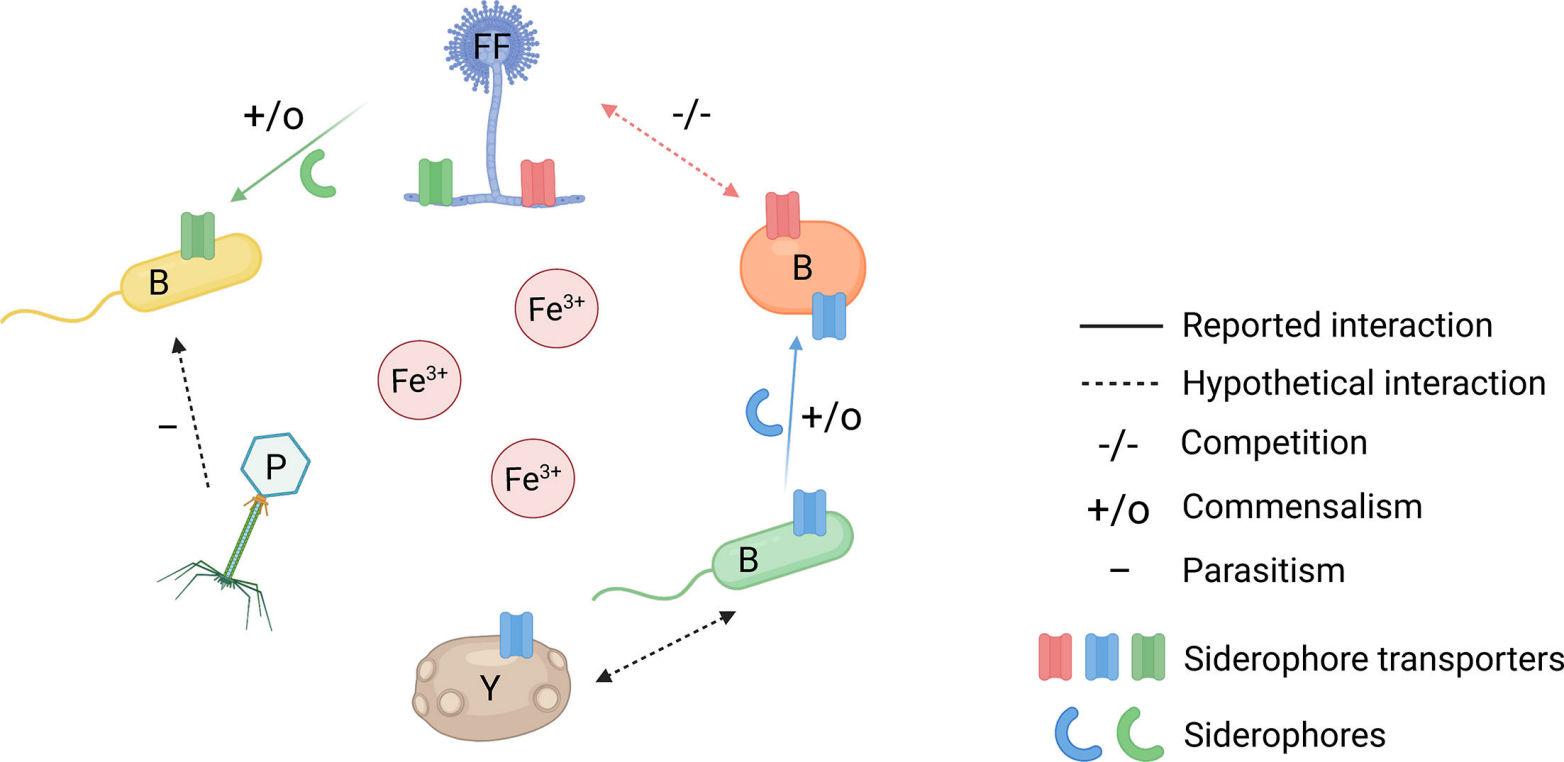
Microbial interactions driven by siderophore-based iron acquisition in surface-ripened cheese. Cheese is an ecosystem in which iron is limiting, and a diverse microbiota, including Gram-positive and Gram-negative bacteria, fungi (yeasts and filamentous fungi), and bacteriophages, makes use of various strategies to acquire iron. Siderophore-mediated iron uptake is a common mechanism. Microbes lacking siderophore biosynthesis pathways but possessing specialized iron transporters can make use of the siderophores produced by others. These interactions drive ecological relationships, including competition for siderophores between microbes carrying the same transporters (competition), passive benefits of siderophores with no impact on the producer (commensalism) (87, 88), and the infection of siderophore producers (parasitism). B, bacterium; FF, filamentous fungus; P, phage; Y, yeast. Created in https://BioRender.com.

Microbial interactions play a major role in shaping the structure and function of the microbiome (81, 82), and the cheese ecosystem is no exception. Microbes frequently compete for iron in the cheese ecosystem due to the limiting amounts of iron present, its poor solubility, and lack of stability (83–85). The production or import of ferric iron-specific chelators known as siderophores is one of the strategies used by cheese microorganisms to overcome iron limitation (86) (Fig. 4).

Monnet *et al.* reported that iron supplementation (1 mg/kg FeCl_3_) in a model cheese system inoculated with a reduced microbial consortium consisting of only three species (*Lactococcus lactis*, *Glutamicibacter arilaitensis*, and *Debaryomyces hansenii*) strongly stimulated the growth of *G. arilaitensis* (89). Moreover, the addition of 1 mg/kg FeCl_3_ and 50 µM desferrioxamine B siderophore to this model cheese system also had a significant impact on surface-ripening bacteria. Iron supplementation enhanced the growth of *Glutamicibacter*, *Corynebacterium*, and *Brevibacterium* strains (83). Our knowledge of the contribution of iron to interactions between bacteria in the presence of fungi within the cheese environment remains limited. Only one research study to date has described the contribution of iron to strong mutualistic interactions between *Hafnia alvei* and *Brevibacterium aurantiacum* through the sharing of siderophores in a model cheese system in the presence of *D. hansenii* (87).

Molecular and genomic approaches can also be used to explore the potential role of iron in the survival of cheese rind microorganisms. Bonham *et al.* reported that 23% of the genes identified in 165 cheese-associated bacteria were involved principally in the acquisition of essential nutrients, including iron, by the bacteria. In the presence of limiting amounts of iron, bacteria produce siderophores for iron scavenging and an iron uptake system for importing iron into the cell. However, some cheese bacteria, including members of *Actinomycetota*, *Bacillota*, and *Pseudomonadota* in cheese, can capture and use siderophores produced by other microorganisms (i.e., xenosiderophore use or siderophore piracy), thereby saving energy (90) (Fig. 4). This selective advantage may be the result of horizontal gene transfer (HGT), a process by which an organism transfers genetic material to another organism that is not its offspring, facilitating adaptation to novel environments (91). For instance, Bonham *et al.* identified a large region of HGT, the iRon Uptake Siderophore Transport Island (RUSTI), as involved in iron acquisition in five different genera of *Actinomycetota* (90). These RUSTI genes can be found in pathogens and cheese *Actinomycetota*, which use similar strategies to acquire iron. For *B. aurantiacum*, one of the most prevalent smear-ripening bacterial species, genome analysis revealed the presence of at least 24 genes involved in iron acquisition and metabolism, including the above-mentioned RUSTI genes shown to be acquired through HGT (92).

### Potential role of iron in bacterial–fungal interactions in the cheese ecosystem

Iron may be a crucial micronutrient in bacterial–fungal interactions in the cheese ecosystem, as Mayo *et al.* highlighted the role of siderophores in a “cross-linking” function during microbial interactions in cheese (27). Cheese rind microbiota have been shown to satisfy their iron requirements principally through the use of siderophores (86, 88, 93). These siderophores may drive within- and between-kingdom interactions between species, with bacteria using fungal siderophores, for example, in various ecological environments, including cheese (94).

To date, a few examples of iron-based interactions between bacteria and fungi have been reported in cheese. Kastman *et al.* showed that fungi promote the growth of a weakly competitive slowly colonizing bacterium, *Staphylococcus equorum*, in cheese, potentially by enhancing iron availability by providing the staphyloferrin B siderophore and free amino acids (88). Cheese-associated filamentous fungi, such as *Penicillium*, can reduce the dependence of cheese bacteria on their own siderophores by providing fungal siderophores, such as ferrichrome and coprogen (86), which greatly increase access to iron. Such mechanisms were demonstrated in a recent study in which five *Penicillium* strains were cocultured with *Staphylococcus equorum. Staphylococcus equorum* displayed a strong transcriptional response in the presence of *Penicillium*, with an upregulation of thiamine biosynthesis, fatty acid degradation, amino-acid metabolism, and a downregulation of siderophore transport genes. *Penicillium* spp. probably consume and break down complex nutrients in the medium, particularly proteins and complex peptides, increasing iron availability. *S. equorum* concomitantly upregulates thiamine biosynthesis, thereby supporting *Penicillium* growth. This metabolic adjustment may represent a trade-off, as *S. equorum* downregulates pathways involved in staphyloferrin B siderophore transport and synthesis (95).

The importance of yeasts in iron and microbial interactions in cheese is currently less clear. However, certain yeasts from dairy products are known to produce siderophores (96), raising the question of the role of yeast siderophores in bacterial–fungal interactions. Computational and coculture research studies have shown that iron may play a vital role in shaping the microbial communities of cheese rind. However, it remains challenging to observe such behavior in complex community interactions in the cheese rind environment.

### Role of iron in cheese spoilage and pathogen development

Many bacterial species require iron for their spoilage and pathogenic activity (97–99). As an environment in which iron is limited, cheese may be an interesting ecosystem in which to investigate the potential role of iron in interactions between pathogenic and spoilage microbes and the desirable cheese microbiota. Many spoilage and pathogenic bacteria use siderophores to obtain iron from food matrices, including cheeses (100). The *Enterobacteriaceae* family includes important species of food-borne pathogens, especially among the genera *Escherichia*, *Salmonella*, and *Shigella* (101, 102). Certain members of this family produce different types of siderophores and have different transport systems (103, 104). *Escherichia coli* secretes enterobactin (105); *Salmonella* produces salmochelin (106); and *Shigella* spp. synthesize either enterobactin or aerobactin (107). Iron availability increases the pathogenic potential of *Salmonella* and other enteric pathogens in the gut ecosystem (108, 109), but there is currently no direct evidence of such an effect in food matrices. Iron availability may also be a key factor modulating the virulence of *E. coli* in the cheese ecosystem. Indeed, Bujňáková *et al.* tested 92 *E. coli* strains for 11 representative virulence genes in Slovakian sheep’s milk cheeses and showed that 30% of the isolates possessing virulence genes also had at least one siderophore gene, potentially enhancing their survival in the cheese environment (110).

*Listeria monocytogenes*, *Pseudomonas* spp., and *Staphylococcus aureus* in cheese may also act as pathogens or spoilage agents (111–113). Genomic surveys have shown that *L. monocytogenes* strains lack the genes required for the biosynthesis of siderophores (114), a finding confirmed physiologically in culture medium (115). However, *L. monocytogenes* can nevertheless use siderophores generated by other organisms as a source of iron (115). *L. monocytogenes* can obtain iron bound to catechol siderophores, such as enterobactin and corynebactin, and hydroxamate siderophores such as ferrichrome, ferrichrome A, and ferrioxamine B (116). Depending on the species producing the siderophore and the type of siderophore produced, siderophore availability may have a positive or negative effect on *L. monocytogenes* growth (117). There is currently no evidence to suggest that *L. monocytogenes* proliferation is linked to iron in the cheese ecosystem. Some strains of *Candida intermedia* and *Kluyveromyces marxianus* yeasts have been shown to inhibit *L. monocytogenes* growth in smear cheese due to competition for nutrients (118). These nutrient-mediated interactions may involve enhanced iron utilization by yeast species.

Some *Pseudomonas* spp. are involved in the spoilage of foods, including cheese (119). *Pseudomonas* spp. can increase their synthesis of extracellular proteases, lipases, and siderophores in iron-deficient environments (120, 121). All these molecules have important functions in cheese. Brown and Luke (122) evaluated the ability of 20 isolates of *Pseudomonas* spp. of known milk spoilage potential to produce siderophores. They found that many of these isolates produced siderophores, principally pyoverdins, and could use siderophores. Some strains of *Pseudomonas* can inhibit the growth of *Listeria monocytogenes*, and, in one strain, this inhibitory potential has been shown to be associated with the production of a compound presumed to be a chromopeptide siderophore (123).

Thus, the activity of certain pathogenic and spoilage bacteria in cheese appears to be related to iron metabolism, including iron acquisition by siderophores. Further studies should focus on deciphering the precise role of iron in the proliferation of these microorganisms to improve control over the risks of consumer infection and food spoilage. Furthermore, an understanding of cheese ecology gained from studies of the interaction between these undesirable and desirable cheese microbiota in the presence of different iron concentrations would provide new insight for better management of cheese safety and quality.

### Role of iron in phage infections

Bacteriophages, also known as phages, are viruses that infect bacteria. In cheese, a number of well-studied phages are known to infect LAB starter cultures, potentially delaying or preventing fermentation (124). In addition, recent metagenomic studies have revealed a wide diversity of uncharacterized phages targeting ripening bacteria, such as species of *Glutamicibacter*, *Brevibacterium*, *Corynebacterium*, *Psychrobacter*, *Leuconostoc*, *Vibrio*, and *Pseudoalteromonas* (125–127). All the dairy phages isolated to date are tailed double-stranded DNA viruses of class *Caudoviricetes* (128–130).

The oxides and salts of iron, like those of many other metals, possess antiviral properties that have been exploited in the development of disinfectants. Iron can directly inactivate bacteriophages (for review, see reference 131) and may also be involved in interactions between phages and bacteria. It has been suggested, for example, that, in environments in which iron is limiting, such as oceans, the iron ions present in the tail fibers of certain phages facilitate the uptake of the phage via the iron-uptake system of the bacterial host, thereby enhancing the ability of the phage to infect its host (132). However, additional studies are required to explore the relevance of this theoretical framework in the cheese ecosystem.

## IRON UTILIZATION AND ITS CONTROL IN CHEESE MICROORGANISMS

Switching from the scale of the ecosystem to that of the individual, we will now focus on microbial metabolism. By following the journey of iron from the extracellular space to the intracellular space, while considering microbial metabolism, we can shed light on the reasons why iron is so important to cheese microbial communities.

### Utilization of intracellular iron

Like microbes from other aerobic environments, cheese-ripening microorganisms likely depend on many iron-requiring enzymes for respiration and oxidation–reduction reactions. Research in this area is limited in ripening microorganisms, but findings from model organisms can help to understand fundamental metabolic processes.

#### Iron storage

After its uptake into the cell, iron is reduced to Fe^2+^, leading to its dissociation from the siderophore complex. When cytoplasmic iron exceeds the needs of the cell, the ions are oxidized and stored in ferritins, bacterioferritins, and Dps proteins for later use in conditions in which iron is limiting. Fe^2+^ is sequestered by these proteins and then oxidized to Fe^3+^, with H_2_O_2_ or O_2_ as electron acceptors (133). In addition to their function in iron storage, these proteins also provide protection against oxidative stress. Between iron transport, storage, and use, a pool of accessible iron ions known as the labile iron pool (estimated concentration of 1–10 µM) remains available for use in the proteome (133). The iron in this labile iron pool is probably Fe^2+^, given the reducing environment of the cytosol.

The combination of aerobic metabolism and reactive Fe^2+^ in the labile iron pool increases the risk of ROS being generated by the Fenton/Haber–Weiss reaction (133, 134). ROS can damage DNA, oxidize protein residues and lipid membranes, and disrupt iron-sulfur (FeS) centers, releasing more free iron into the cell. Bacteria protect themselves against ROS damage by producing detoxifying enzymes. Superoxide dismutases (SODs) act on the superoxide anion, generating H_2_O_2_ or O_2_. Catalases and peroxidases subsequently deactivate H_2_O_2_ (135). Some SODs (FeSOD), peroxidases, and catalases are iron dependent (136). Additionally, there have been reported cases that iron limitation also induces an oxidative stress response (137–139).

#### Iron proteome

The substantial incorporation of iron into the bacterial proteome has been attributed to its ability to both donate and accept electrons. Its two redox states, Fe^2+^ and Fe^3+^, enable iron to participate in numerous pathways through incorporation into enzymes or involvement in electron transfer processes (56).

Intracellular iron from the labile iron pool is directed toward storage proteins, cofactor assembly, and the transport of cofactors to apoproteins. Iron is incorporated into the proteome through assembly into FeS clusters, heme, or in its elemental form. The assembly of iron into FeS clusters and heme is complex, requiring the activity of several enzymes (140). FeS cluster assembly is conserved across all domains of life. Several biosynthesis systems exist and are known to have evolved independently. The relevant biogenesis systems for ripening bacteria are the iron-sulfur cluster (ISC) assembly and sulfur mobilization. Both these systems can be present in the same cell. Their mechanisms of action differ, but both include a sulfur donor, an electron donor, iron, the assembly of the FeS cluster on a scaffold, and transport to the targeted apoprotein (141).

Heme iron also interacts with the proteome. There are three pathways of heme synthesis in bacteria that are dependent on coproporphyrin, protoporphyrin, and siroheme. The use of heme by the microbe depends on its form. Hemes a, b, c, d, and o are used in the electron transport chain (ETC) during aerobic respiration. However, hemes b and c have broader roles extending beyond aerobic respiration to anaerobic respiration, other types of electron transfer, and as cofactors for various enzymes (142).

These cofactors bind to a substantial portion of the microbial proteome through roles in aerobic and anaerobic respirations, enzymatic anabolic and catabolic conversions, oxidative stress management, DNA synthesis and repair, and in transcriptional and posttranscriptional modifications (141, 142).

The coordination of all these intricate processes—from iron acquisition to defense against ROS and the assembly of iron cofactors—requires very tight regulation of iron metabolism. These processes satisfy the fundamental iron requirements of most (aerobic) microorganisms, including those involved in cheese ripening, and explain how iron can shape both the composition of microbial populations and their interactions.

### Transcriptional and posttranscriptional regulation

Iron homeostasis and the regulation of metabolism are controlled by a highly conserved mechanism involving the ferric uptake regulator (Fur). Fur can act as both a repressor or an activator. In its holo-form, it acts as a repressor by binding to the promoter region (133, 143, 144). However, holo-Fur can also function as an activator in some microbes (145). Furthermore, apo-Fur has been shown to fulfill both functions in certain microorganisms (146). With its multifaceted mode of action, Fur may regulate the transcription of over 100 genes (133). In Gram-positive bacteria with a high GC content, like many of the *Actinomycetota* on the cheese surface, diphtheria toxin repressor/iron-dependent regulators also act as iron-dependent global regulators, functioning in a similar manner to Fur (133, 147).

The Fur regulon is also involved in the posttranscriptional modification of enzymes involved in iron metabolism. *E. coli*, which has been extensively studied, has been shown to use the non-coding sRNA RyhB to regulate many iron-containing enzymes. It serves as a type of hierarchical selection on enzymes that have functionally equivalent counterparts, usually proteins that can use another metal as a cofactor, limiting iron use to the proteins for which iron is strictly required (136, 148). For example, it mediates the replacement of the Fe-requiring SOD by MnSOD, the replacement of the Fe-requiring ribonucleotide reductase (RNR) by a Mn-requiring RNR, and the repression of succinate dehydrogenase (SDH) as a result of mRNA degradation (136).

Aconitase, another iron-requiring tricarboxylic acid (TCA) cycle enzyme regulated by RyhB, is a dual-function protein. Under conditions of iron limitation, the apo-form adopts the function of a regulatory RNA-binding protein capable of binding to its own *acnB* mRNA to inhibit degradation. Interestingly, citrate, the substrate of aconitase, prevents the switch from holo- to apo-AcnB. Thus, if iron concentration is low, but citrate concentration is high; AcnB expression levels decrease, whereas if both iron and citrate concentrations are low (more apo-AcnB), this enzyme adopts the function of a regulatory RNA-binding protein. This results in a sophisticated, layered posttranscriptional regulation of essential iron-requiring enzymes that is dependent on the intracellular iron pool (149).

Similar RyhB-like mechanisms have been studied in other microorganisms: FsrA in *Bacillus subtilis*, Cth2 in *S. cerevisiae*, and PrrF in *Pseudomonas aeruginosa* (136, 150–152). There may be other similar regulatory systems in cheese-ripening microorganisms, but these are currently understudied and a target for future research.

## CHANGES IN CENTRAL AND SECONDARY METABOLISMS IN RESPONSE TO IRON LIMITATION

### Interplay between the TCA cycle and the pentose phosphate pathway

The microorganisms growing on the surface of the cheese are aerobic. They therefore use the TCA cycle and oxidative phosphorylation for ATP generation. The TCA cycle includes several enzymes with FeS clusters, and iron limitation may cause its disruption, causing a shift in metabolism. The pentose phosphate pathway (PPP) is one possible alternative metabolic route. Aconitase and SDH require FeS clusters for their catalytic activity. Aconitase binds a [4Fe-4S] cluster but may also be present in its apo form. Succinate dehydrogenase binds three FeS clusters: [2Fe-2S], [3Fe-4S], and [4Fe-4S] (153). This enzyme takes part in the TCA cycle and complex II of the ETC. In total, 12 FeS clusters are present in ETC complexes I, II, and III, three of which are in complex II–SDH (154). Cytochromes with heme centers also participate in the ETC in complexes II, III, and IV (155). Together, they drive the transfer of electrons through the ETC, which is the chief source of ATP in aerobic microorganisms. A partial loss of aconitase activity due to iron starvation could have notable implications for the TCA cycle and SDH activity.

Activation of the iron-starvation response has been shown to lead to the accumulation of citrate, malate, and fumarate in the cell, whereas 2-oxoglutarate was present at lower levels (156–158). Citrate is a known allosteric inhibitor of phosphofructokinase (PFK), a glycolytic enzyme that catalyzes the conversion of fructose 6-phosphate to fructose 1,6-biphosphate (159). If PFK is inhibited by citrate under conditions of iron limitation, the carbon flux can be re-directed toward the PPP, as demonstrated in *E. coli* and *S. cerevisiae* (160, 161). The PPP is the main source of NADPH generation in cells (159). NADPH is involved in the biosynthesis of amino acids, fatty acids, and deoxyribonucleotides. Amino-acid biosynthesis is particularly closely connected to the PPP pathway. The synthesis of almost all amino acids depends either on NADPH, glutamine, glutamate, PPP precursors, or a combination of these molecules. This is particularly evident in the *de novo* synthesis of methionine, which plays an important role in ripening due to its involvement in volatile sulfur compound pathways (11). NADPH also contributes to anti-ROS defense by regenerating glutathione (162, 163). Moreover, it serves as a reductant in the ISC machinery for FeS biogenesis (141).

Iron limitation may also affect the TCA cycle through activation of the glyoxylate shunt (GS). Microorganisms in aerobic environments with low iron levels resembling those of cheese have been reported to bypass steps of the TCA cycle, re-directing the carbon flux toward the GS (138, 164, 165). This is the case in marine bacteria and pathogens. The GS shunt bypasses the CO_2_-producing steps of the TCA cycle by converting the six-carbon isocitrate into glyoxylate (2C) and succinate (4C) in a reaction catalyzed by isocitrate lyase. Malate is then generated from glyoxylate and acetyl-CoA and converted to oxaloacetate for entry into gluconeogenesis (159). Koedooder *et al.* have suggested that the GS is activated to direct carbon flux away from the Fe-heavy ETC (138).

The exploration of these metabolic pathways in microorganisms involved in cheese ripening would make it possible to determine whether the disturbances of the TCA cycle caused by iron deficiency affect the production of secondary metabolites and, therefore, the organoleptic characteristics of cheese. This is particularly important, given that metabolic processes related to amino-acid metabolism, especially for sulfur-containing amino acids such as methionine, and fatty acid metabolism are linked to NADPH requirements and the production of aromatic compounds in cheese (11, 163). It should be stressed, however, that these hypotheses have merely been inferred from microorganisms living in similar environments. A comprehensive study of these pathways in the cheese environment is required to evaluate their relevance in ripening microorganisms.

### Interplay between the TCA cycle and amino-acid metabolism

The impact of iron limitation on the TCA cycle can have various consequences for other metabolic pathways, such as amino-acid metabolism. During ripening, the LAB deplete the medium of lactose, which is therefore unlikely to be the main energy source for most ripening microorganisms. Yeasts, such as *K. lactis*, *K. marxianus*, and *D. hansenii*, also use the residual amounts of lactose present at the start of the ripening process. At later stages, lactate, proteins, and lipids become the main energy sources for ripening microorganisms, especially aerobic bacteria that predominate during this period in smear cheeses (34). Cow’s milk contains about 3.0%–3.9% protein, which can be used as an energy source by ripening microorganisms with proteolytic activity (34). Extracellular proteases may also facilitate cross-feeding in microbial populations (87). The amino acids released by protein hydrolysis may undergo a series of reactions leading to their direct or indirect entry into the TCA cycle for ATP production. These pathways may be particularly important under conditions of iron limitation, when there is not enough iron to support TCA cycle reactions. Amino acids, such as glutamate, the most abundant amino acid in caseins, aspartate, and alanine, follow a more straightforward pathway, yielding α-ketoglutarate, oxaloacetate, and pyruvate through transamination. Other amino acids, such as branched-chain amino acids (BCAA), have to go through transamination to produce α-keto acids, followed by several enzymatic steps to yield acetyl-CoA and succinyl-CoA for entry into the TCA cycle (166, 167). In conditions of iron limitation, these pathways can replenish TCA cycle intermediates through anaplerotic pathways. Anaplerosis is the replenishment of depleted TCA cycle intermediates to ensure a partial maintenance of TCA cycle function (166).

Microbes can manage the carbon load generated by anaplerotic reactions (or bottlenecks induced by iron limitation) by using glycolysis and TCA cycle intermediates for the synthesis of various compounds via cataplerosis. For instance, amino-acid carbon skeletons can be obtained by glycolysis: valine and leucine from pyruvate, glycine and cysteine from 3-phosphoglycerate, and aromatic amino acids from chorismate. The amino group is obtained from a nitrogen-containing entity, often ammonium, produced during glutamate synthesis (159). Transamination then transfers the amino group to other carbon skeletons to form other amino acids.

Studies of the interaction between amino acids and the TCA cycle could reveal whether the synthesis or breakdown of amino acids in the context of the TCA cycle influences the sensory attributes of cheese. This is particularly relevant for cataplerotic reactions, as amino acids can undergo further degradation to produce aromatic compounds. Conversely, anaplerotic reactions could be used as a strategy to ensure the maintenance of carbon flux through the TCA cycle. In addition to its importance for energy metabolism, the TCA cycle supplies important intermediates for the synthesis of siderophores.

### TCA cycle intermediates as siderophore precursors

Various metabolic intermediates can serve as structural components of siderophores. In addition to citrate and amino acids, α-ketoglutarate and succinyl-CoA have been shown to act as building blocks for the biosynthesis of some siderophores (62). Achromobactin is synthesized from citrate, serine, and α-ketoglutarate; aerobactin is synthesized from lysine and citrate; petrobactin and legiobactin are synthesized from citrate; alcaligin and desferrioxamines are synthesized from lysine and succinyl-CoA; staphyloferrin B is synthesized from citrate and α-ketoglutarate; enterobactin is synthesized from serine and (glycolytic) chorismate; pyochelin is synthesized from (glycolytic) chorismate and cysteine; pyoverdines are synthesized from several amino acids and, sometimes, succinate, α-ketoglutarate, and malate; and coprogen and ferrichrome are synthesized from ornithine (ornithine biosynthesis pathway involving α-ketoglutarate and glutamate) (59, 62, 63, 168). As summarized in Table 1, a few of these siderophores can be synthesized by bacteria isolated from the surface of cheese. The IucA/IucC domain, present in many ripening bacteria (Table 1), is conserved within NIS siderophores, which are typically generated from citrate or succinate derivatives as precursors (59). These findings highlight the need for a maintenance of carbon flux through the TCA cycle, possibly via anaplerosis.

In *S. aureus*, the *sbn* gene cluster ensures staphyloferrin synthesis even if the TCA cycle is slowed down (169). The TCA cycle enzyme citrate synthase has been reported to be downregulated in conditions of iron limitation (170, 171). In such instances, SbnG acts as a second citrate synthase, catalyzing citrate synthesis from oxaloacetate and acetyl-CoA. Moreover, SbnA and SbnB have been shown to catalyze the synthesis of α-ketoglutarate from L-glutamate. These pathways serve as an alternative source of replenishment for staphyloferrin B precursors that is independent of the TCA cycle and not repressed by the iron-sparing response (169). The cheese-ripening bacterium *S. equorum* subsp. *equorum* Mu2 (National Center for Biotechnology Information RefSeq assembly: GCF_000297455.1) probably makes use of a similar mechanism as it has *sbnA* and *sbnB* genes and a putative *sbnG* gene.

A metabolic flux analysis with labeled carbon in *Pseudomonas* revealed that gluconeogenic substrates are preferred over glycolytic substrates in conditions of iron limitation. Cells combat iron limitation to sustain the production of TCA cycle-derived precursors for siderophore synthesis by re-routing their metabolism through anaplerotic pathways. This re-routing ensures a continuous supply of key intermediates of carbon metabolism, supporting the production of precursors for synthesis of the siderophores required for iron acquisition (172).

Similar analyses of ripening microorganisms would provide us with a deeper understanding of siderophore synthesis and the metabolic changes induced by iron limitation. The strategies used may include an increase in the production of relevant TCA cycle-derived precursors, for use as direct intermediates or as compounds involved in anaplerosis, such as amino acids.

### Amino-acid biosynthesis under conditions of iron limitation

As indicated above, proteins can serve as an energy source during cheese ripening. However, if the amino-acid supply is exhausted, these molecules must be synthesized by prototrophic microbes. The activity of several enzymes involved in amino-acid synthesis requires FeS clusters. The biosynthesis of BCAAs, in particular, is dependent on FeS cluster enzymes. Dihydroxy-acid dehydratase (IlvD) is involved in all three BCAA pathways, and isopropylmalate isomerase (LeuC) is involved in leucine biosynthesis. In the cheese-ripening fungus *G. candidum*, analyses in synthetic medium revealed an accumulation of 2-isopropyl malate (substrate of LEU1, homolog of LeuC). The authors suggested that LEU1 activity was low due to the limiting levels of iron in cheese (173).

Similarly, in *S. cerevisiae*, ILV3 and LEU1 (homologs of IlvD and LeuC) are downregulated, and 2-isopropyl malate accumulates in large quantities in a medium in which iron is limiting. However, these effects are not reflected in the metabolic pool, as no decrease in amino-acid levels was detected. On the contrary, the total pool of BCAA increased. The authors suggested that despite the downregulation of BCAA synthesis genes, FeS enzymes were present in excess in conditions of iron sufficiency such that no decrease in enzyme activity levels occurred in conditions of iron limitation (156).

In *Corynebacterium glutamicum*, amino acids were reported to accumulate in conditions of iron starvation, induced by the low pH of the medium. The authors reported increases in the levels of several amino acids, including BCAAs and cysteine and methionine pathway products. Pyruvate accumulated at levels 11 times higher, possibly due to improper functioning of the TCA cycle, leading to the synthesis of BCAAs (157).

These findings highlight the importance of amino acids in maintaining cellular functions, possibly combating iron limitation. Despite the iron requirements of several enzymes involved in the biosynthetic pathway for amino acids, the metabolic pool of amino acids is not necessarily smaller in conditions of iron limitation. The changes in methionine and cysteine metabolism are particularly important and merit further investigation. These observations may have major implications for cheese-ripening microorganisms, given the involvement of these pathways in the production of volatile sulfur compounds.

### Pigment formation

Smear-ripened cheeses such as Epoisses, Livarot, Munster, Tilsit, and Romadour are widely consumed in Europe. They develop an orange-red surface color during ripening due to the presence of certain pigment-producing microorganisms. The red-orange smear is predominantly attributed to members of *Actinomycetales*, namely, *Brevibacterium linens*, *B. aurantiacum*, *Microbacterium gubbeenense*, and *G. arilaitensis* (174). Carotenoids are the predominant pigments typically found in cheese rinds. The aromatic pigments isorenieratene and its derivatives 3-hydroxy-isorenieratene and 3,3′-di-hydroxy-isorenieratene are associated with the presence of *B. linens* and *B. aurantiacum* and are produced through arylation and hydroxylation (175). In *B. aurantiacum*, the heme-containing cytochrome P450-dependent monooxygenase hydroxylates isorenieratene, indicating that iron availability may therefore affect the metabolic pathways leading to pigment formation (174). A recent study on changes to cheese microbial communities caused by iron fortification reported an increase in pigment formation on the surface of iron-fortified mini-model cheeses (176). Pigment formation is an essential element of the sensory properties of smear-ripened cheeses. Iron appears to influence this process, and its contribution requires further study.

## CONCLUSIONS

Iron can drive microbial interactions in various ecosystems, including cheese. The cheese ecosystem harbors a diverse but moderately complex microbiota that may provide researchers with a promising *in situ* system for investigations of both iron metabolism and the mechanisms of iron-driven interactions. The cheese microbiota is uniquely successful in a highly competitive environment subject to iron limitation, and studies of this microbiota can provide critical insight into the mechanisms of iron acquisition, utilization, and regulation. A combination of multiomics approaches would help to decipher the strategies by which the cheese microbiota overcomes the challenges of iron limitation, revealing key adaptations, such as siderophore biosynthesis, iron transport mechanisms, and metabolic shifts (176). These findings would improve our understanding of the role of iron in cheese ecology and would have broader implications for our understanding of microbial interactions in other ecosystems. They would also improve the selection of microorganisms with desired functions in cheese, such as better organoleptic properties, quality, and food safety (5, 7).

However, there are likely to be several obstacles to advances in this field of research. First, advances would be required in the chemical and structural characterization of siderophores and their transporters, together with identification of the genes involved (177, 178). Such advances would facilitate large-scale *in silico* analyses. Second, iron metabolism has been overlooked in fungi compared to bacteria (179), and further studies in fungi are therefore required. Little is known about the role of yeasts in iron-based microbial interactions in cheese, but also in other ecosystems. Many fungal siderophores have yet to be described and characterized. Finally, alternative non-siderophore iron acquisition systems involving other molecules, such as lactoferrin (180) or heme (181), require further investigation to shed light on their potential contribution and importance to microbial iron metabolism in cheese and other ecosystems.

## Data Availability

The scripts and data used to produce the figures are available at https://entrepot.recherche.data.gouv.fr/dataverse/cheese_iron_metabolism.
